# “Minimal clinically important difference” estimates of 6 commonly-used performance tests in patients with chronic musculoskeletal pain completing a work-related multidisciplinary rehabilitation program

**DOI:** 10.1186/s12891-018-2382-2

**Published:** 2019-01-05

**Authors:** Charles Benaim, Simon Blaser, Bertrand Léger, Philippe Vuistiner, François Luthi

**Affiliations:** 1 Department of Physical Medicine and Rehabilitation, Orthopaedic Hospital, Lausanne University Hospital, Av Pierre Decker 4, CH-1011 Lausanne, Switzerland; 2 Institute for Research in Rehabilitation, Clinique Romande de Réadaptation, Sion, Switzerland; 3 Department of Medical Research, Clinique Romande de Réadaptation, Sion, Switzerland; 4 Department for Musculoskeletal Rehabilitation, Clinique Romande de Réadaptation, Sion, Switzerland

**Keywords:** MCID, Functional tests, Chronic musculoskeletal pain

## Abstract

**Background:**

Functional tests are widely used to measure performance in patients with chronic musculoskeletal pain. Our objective was to determine the Minimal Clinically Important Differences (MCID) for the 6-min walk test (6MWT), the Steep Ramp Test (SRT), the 1-min stair climbing test (1MSCT), the sit-to-stand test (STS), the Jamar dynamometer test (JAM) and the lumbar Progressive Isoinertial Lifting Evaluation (PILE) in chronic musculoskeletal pain patients.

**Methods:**

A single-center prospective observational study was conducted in a rehabilitation center. Patients with upper-limb, lower-limb or neck/back lesions were included over a period of 21 months. We used the anchor-based method as a reference method, supplemented by the distribution-based and opinion-based approaches, to determine the MCIDs.

**Results:**

838 chronic musculoskeletal pain patients were included. The estimation method and thelesion location had a significant influence on the results. MCIDs were estimated at +75m and +60m for the 6MWT (lower-limb and neck/back lesions, respectively), +18 steps for the 1MSCT (lower-limb and neck/back lesions) and +6kg for the JAM (upper limb lesions). The anchor-based method could not provide valid estimations for the three other scales, but distribution and opinion-based methods provided rough values of MCIDs for the SRT (+39w to +61w), the STS (-5 sec to -7 sec) and the PILE (+4kg to +7kg).

**Conclusion:**

The above MCID estimations for the 6MWT, 1MSCT and JAM can be used in chronic musculoskeletal pain patients participating in vocational multidisciplinary rehabilitation programs or in therapeutic trials. The use of specific anchors might give better estimations of MCIDs for the three other scales in future research.

## Background

The prevalence of chronic (more than 3 months) musculoskeletal pain is higher than 20% in industrial countries [1]. It contributes to physical disability [2] and, according to Swiss federal statistics [3], costs from $6 to 14 billion a year in Switzerland (11 to 25% of the Gross Domestic Product). Evaluations of pain and functional status are major issues for chronic musculoskeletal pain patients. Many performance tests are currently being used by clinicians, especially in rehabilitation centers. Among these tests, the 6-min walk test (6MWT) [4], the Steep Ramp Test (SRT) [5], the 1-Minute Stair Climbing Test (1MSCT) [6], the Sit-To-Stand test (STS) [7], the Jamar dynamometer test (JAM) [8] and the lumbar protocol of the Progressive Isoinertial Lifting Evaluation (PILE) [9] are widely used in patients with musculoskeletal, neurological, cardiac or pulmonary disease. These tests are easy to administer and do not require expensive devices.

In clinical research, small improvements in patient-reported outcomes may be statistically significant, but clinically irrelevant. The concept of “Minimal Clinically Important Difference” (MCID) was introduced by Jaesche et al. in 1989 to study the clinical importance of such improvements [10]. The MCID of a clinical scale is very useful to know, as it is the smallest change in an outcome that a patient would identify as important. MCID values can of course be used by clinicians in daily clinical practice, but are also helpful for research purposes. For example, the MCID can be used to estimate the number of subjects required in a therapeutic trial. MCIDs of the 6MWT have been estimated for patients with coronary artery disease [11], chronic obstructive pulmonary disease [12] and Duchenne muscular dystrophy [13], but has never been estimated in chronic musculoskeletal pain patients. Similarly, apart from the PILE which was evaluated in patients with low back pain completing a functional restoration program [14], the MCID of the other tests was not evaluated in chronic musculoskeletal pain patients.

Three general approaches are currently used to determine the MCID of a clinical scale [15]: anchor-based methods, distribution-based methods and opinion methods.

*- In the anchor-based method*, the change in the outcome measure is compared with a subjective anchor variable completed by the patient. For example, patients are asked to rate their perceived global evolution from before to after the treatment with a 7-level Global Rating of Change (GRC) from − 3: “much worse”, to + 3: “much better” [16]. The binary anchor variable is then compared with the outcome measure using ROC curve techniques [17]. Additionally, the “credibility” of the anchor is settled if its correlation with the change in the clinical scale is higher than 0.3–0.4 [18, 19]. Otherwise, the anchor should be considered inappropriate for establishing the MCID. For some authors, this method is the best because it relies on the patient’s subjective assessment [19–22]. For this reason, it was considered as the reference method in the present work.

*- In the distribution method*, the change in the outcome score is compared with a measure of variability. For example, the Standard Error of Measurement (SEM) is the score’s variation due to unreliability. The distribution method is useful to evaluate in cases where the anchor technic is inappropriate or produces lower MCID values. The MCID should be at least equal to the SEM [23].

*- The opinion-based approach* consists in gathering opinions of experts (or patients), using an iterative consensus approach like the Delphi method (see an example in [24]).

Our main objective was to determine MCIDs for the 6MWT, PILE, SRT, 1MSCT, STS and JAM in chronic musculoskeletal pain patients presenting with upper-limb, lower-limb and neck/back lesions and undergoing a multidisciplinary functional rehabilitation program. A secondary objective was to compare MCIDs using the opinion-based methods with those obtained using the reference Anchor-based and the distribution-based approaches.

## Methods

### Study design and setting

A single-center prospective study was conducted in a 140-bed rehab center in the French-speaking part of Switzerland. All rehab inpatients of working age (18–65) and admitted for chronic musculoskeletal pain between May 2014 and January 2016 after an acute orthopedic injury of neck/back, upper limb or lower limb were considered for inclusion.

### Participants

Inclusion criteria were as follows: French-speaking working-age patients, able to understand and sign the study informed consent form, able to answer the questionnaires used in the study, suffering from chronic musculoskeletal pain (at least 3 months). Patients were excluded from the study if they met any of the following criteria: under legal custody (whatever the reason), more than one of the three defined lesion locations, severe traumatic brain injury at the time of the accident if any (Glasgow coma Scale ≤8), spinal cord injury, injury severity graded ≥3 according to the Abbreviated Injury Scale (1 = minor, 2 = moderate, 3 = serious, 4 = severe, 5 = critical, 6 = maximal, currently untreatable) [25]. The study was approved by the local ethics committee (CCVEM 034/12). All participants signed an informed consent form before enrolment.

### Rehabilitation program

During their stay in the rehabilitation clinic, all patients received four to six therapy sessions a day, 5 days a week for 4–5 weeks. Sessions included physiotherapy (individual sessions), physical reconditioning (class-based strengthening, balance and stretching), occupational therapy, vocational therapy and cognitive-behavioral therapies.

### Functional tests

The following functional tests are part of the systematic assessment made at intake and discharge in our rehab center. They all are easy to administer and have good psychometric properties.

*- The 6MWT* measures the distance an individual is able to walk as quickly as possible in a total of six minutes on a flat surface [4].

*- The SRT* is an incremental graded exercise test on a cycloergometer (70–80 rpm), were the load increases by 25 watts every 10 s until the patient’s exhaustion. The test ends when the pedal frequency falls under 60 rpm [5].

*- The 1MSCT*: The patient is invited to walk up and down stairs (one step at a time) for 1 min, the score being the total number of steps climbed [6].

*- The STS*: The patient sits in a chair with his back against the back of the chair, and is asked to stand up straight as quickly as possible five times, without stopping in between. The score is the time required to complete the test [7].

*- The Jamar Handgrip Dynamometer* measures the maximum isometric grip strength. The dynamometer is set to the second handle position and the test is performed three times with both hands; the score is the average of the six measurements in kg [8].

*- The lumbar PILE* involves lifting weights placed in a box from the floor to waist height (0-76 cm), four times in 20 s. The weight is increased incrementally by 2.5 kg/5 kg (women/men), starting with 5 kg/7.5 kg (women/men). The test is terminated (1) by the subjects themselves (fatigue, excessive discomfort or inability); (2) by the examiner if the subject’s heart rate reaches 85% of the age-determined maximum heart rate; or (3) when the subject reaches the limit of 55 to 60% of his body weight [9].

Patients with neck/back and lower-limb lesions performed the 6MWT, SRT, 1MSCT and STS, patients with upper-limb lesions performed the JAM, and all of the patients performed the PILE.

All functional tests were performed at admission and at discharge (length of stay 31 ± 10 days).

### Other data collected

#### At intake

General and sociodemographic variables (sex, age, educational level, length of stay, duration of symptoms), pain location (neck/back, upper limb, lower limb), Abbreviated Injury Scale, the Brief Pain Inventory [26], the Hospital Anxiety and Depression Scale [27]. The educational level was split in “≤ 9 years” and “> 9 years”, which corresponds to the compulsory school duration in Switzerland (before the most recent reform, which set this duration at 11 years for the start of the 2015/2016 academic year).

#### At discharge

The 7-level (GRC) from − 3: “much worse”, to + 3: “much better”. All patients were specifically asked to answer the GRC with respect to the injury they were hospitalized for.

### Data collection and bias

Data at entry were collected before starting the therapeutic program. Records were collected electronically with a digital pen. Functional tests were done under the supervision of highly experienced physiotherapists, familiar with the tests through clear instructions and with chronic pain through regular training: 73% of our physiotherapists have received specific training in chronic pain management; regular complementary training seminars are organized at the clinic (at least twice a year) in addition to weekly multidisciplinary meetings in each hospitalization unit.

### Estimation of the MCIDs

In addition to the ROC reference technique, we calculated the SEM in cases where the ROC was not conclusive. The Delphi process was also used to fulfill the secondary objective.

#### Anchor-based method

Evidence supports the use of a 7 to 11-point numerical scale [28] and the experience we have of our patients supports the use of a 7-point scale rather than a 9 or a 11-point scale. The ROC technique was therefore ran with a 7-level GRC as the subjective anchor. Patients in rehabilitation services are generally treated intensively and for several weeks (4–5 weeks in our clinic). For this reason, we considered that a “slight” improvement (GRC = + 1) was not clinically significant given the multidisciplinary resources invested during the stay. In line with previous works, we considered as a “significant improvement” GRC scores ≥ + 2 [29, 30]. Correlations between GRC scores and changes in functional scores were calculated in order to check the “credibility” of the GRC as an anchor [19]. Also were calculated the correlations between GRC scores and changes in the Brief Pain Inventory and the Hospital Anxiety Depression Scale.

#### Distribution-based method

The SEM was calculated as the square root of the mean square error term from a repeated-measure ANOVA (intake and discharge), which has the advantage of not being influenced by systematic error (in this study, the effect of the therapeutic program between intake and discharge), compared to other methods that rely on the Intraclass Correlation Coefficient [31]. Since an improvement was expected for every patient between admission and discharge, we did not penalized the SEM for systematic difference, which would be due to the effect of the intervention, and not to a lack of reliability.

#### Opinion-based method

The Delphi process was conducted with 19 experts (5 physicians, 9 physiotherapists and 5 occupational therapists, having at least 5 years of experience in musculoskeletal rehabilitation). Three rounds were organized: (1) gathering MCID values of each expert for the 6 scales; (2) 2nd estimation, knowing the 1st round anonymous results (mean-median-min-max for each scale); (3) 3rd estimation, knowing the 2nd round anonymous results and the arguments given by experts who gave the lowest and highest values for each scale.

In the anchor-based and the distribution-based methods, different estimations of the MCID were made separately for upper-limb, lower-limb and neck/back lesions. As the SEM is the score’s variation due to unreliability, MCIDs smaller than the SEM were not considered valid. In the opinion-based method, a single estimation was made for each functional test. All statistical analyses were performed using NCSS 9 [32].

## Results

### Participants

**Fig. 1:**
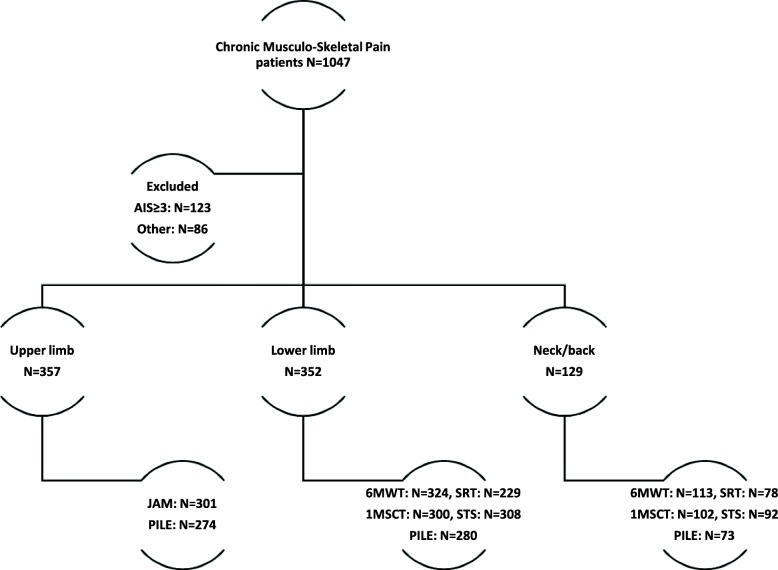
Flow chart of participants who performed functional tests both at intake and at discharge. AIS: Abbreviated Injury Scale. N: number of patients. 6MWT: 6-Minute Walk Test, SRT: Steep Ramp Test, 1MSCT: 1-Minute Stair Climbing Test, STS: Sit-To-Stand test, JAM: Jamar dynamometer test, PILE: Progressive Isoinertial Lifting Evaluation

Between May 2014 and January 2016, one thousand and forty-seven patients were admitted to the clinic for the management of chronic pain after trauma to a limb or spine, 838 patients were eligible for inclusion. Their baseline characteristics, GRC and functional scores at admission and discharge are reported in Tables 1 and 2. The vast majority of our patients have had a bruise, a sprain or a simple fracture of a limb or spine. A flow chart with the number of patients assessed at intake and at discharge is presented in Fig. 1. Depending on the lesion location and the functional scale, 57–92% patients had both assessments.

**Table 1:** Baseline characteristics of patients

Characteristics	*N*	Values
Age	838	44 ± 11
Sex	838	735 (88%) male
Educational level	835	
- ≤ 9 years		487 (58%)
- > 9 years		348 (42%)
Lesion location	838	
- Neck/back		129 (15%)
- Upper limb		357 (43%)
- Lower limb		352 (42%)
AIS	818	
- Minor		304 (37%)
- Moderate		514 (63%)
Duration of symptoms	837	Median [IQ]: 401 days [257–722]
Length of stay	838	31 ± 10
GRC	825	
- Improved (GRC ≥ + 2)		280 (34%)
- Not improved (GRC < + 2)		545 (66%)

Values are presented as Mean(±Standard Deviation) or N(percentages), unless stated otherwise. Age in years, Length of stay in days. *AIS* Abbreviated Injury Scale, *GRC* Global Rating of Change. *N* number of patients

**Table 2:** Functional scores and questionnaires at admission and at discharge

Functional scores	*N*	Admission	Discharge	*p*
6MWT	437	427 ± 127	492 ± 132	< 10^− 6^*
SRT	307	168 ± 88	204 ± 94	< 10^− 6^*
1MSCT	402	83 ± 37	100 ± 42	< 10^− 6^*
STS	400	19 ± 17	15 ± 13	< 10^−6^*
JAM	301	22 ± 15	26 ± 16	< 10^−6^*
PILE	627	16 ± 9	18 ± 9	< 10^−6^*
Questionnaires	N	Admission	Discharge	*p*
BPI	764	10 ± 4	9 ± 4	< 10^−6^*
HADS	803	18 ± 8	17 ± 9	0.004*

Values are presented as Mean(±Standard Deviation). *N* number of patients. 6-Minute Walk Test (6MWT) in meters, Steep Ramp Test (SRT) in Watts, 1-Minute Stair Climbing Test (1MSCT) in steps, Sit-To-Stand test (STS) in seconds, Jamar dynamometer test (JAM) in Newtons, Progressive Isoinertial Lifting Evaluation (PILE) in kg. *BPI* Brief Pain Inventory, *HADS* Hospital Anxiety and Depression Scale. *Statistically significant

Seven hundred and thirty five were male (88%), mean age was 44 years ±11, median duration of painful symptoms was > 1 year (401 days). Mean length of stay in the rehabilitation clinic was 31 days±10, 280 (34%) declared a significant improvement during the stay, as defined by a GRC between + 2 and + 3.

### MCID - anchor-based method

For each functional test and each lesion location, one ROC curve was drawn with the GRC as the condition variable and the score progression (intake-discharge) as the criterion variable. The optimal cutoff on the ROC curve was determined as the value that gave the highest Youden index (sensitivity+specificity-1), provided that both sensitivity and specificity were simultaneously ≥60%. Only MCIDs for the 6MWT, 1MSCT and JAM reached this condition (Table 3). As an example of inconclusive estimation, the value of + 2.5 kg for the PILE gave 0.66/0.56 (good sensitivity/insufficient specificity) and + 5.0 kg gave 0.46/0.73 (insufficient sensitivity/good specificity), in patients with upper limb lesions. Therefore, we could only state that a hypothetic MCID value for the PILE could be estimated between + 2.5 and + 5.0 kg, depending on whether we prioritize the sensitivity or the specificity (+ 0.0 to + 2.5 kg in lower limb lesions, 2.5 kg to 5.0 kg in neck/back lesions). In the same way, we found for the STS -2.0 to − 3.3 s (lower limb) and − 4.0 to − 6.0 s (neck/back), and for the SRT + 25 to + 55 W (lower limb) and + 25 to + 50 W (neck/back). The corresponding “area under the ROC curve”, which illustrated the test’s performance as a binary classifier (“improvement”/“no improvement”), ranged from 0.65 to 0.72. GRC scores of patients with lower-limb and neck/back lesions correlated with changes in the 6MWT and 1MSCT scores (Table 4); GRC scores of patients with upper-limb lesions correlated with changes in the JAM and PILE scores. All of these correlations reached the recommended value of 0.3–0.4 [23, 24]. GRC scores also correlated significantly with changes in the Brief Pain Inventory (*r* = − 0.40, *p* <  10^− 6^) and the Hospital Anxiety and Depression Scale (*r* = − 0.22, *p* <  10^− 6^). It was not influenced by age or sex.

**Table 3:** Results of the Anchor-based, Distribution and Opinion methods

	Anchor-based method/Distribution method			Opinion
	Upper limb	Lower limb	Neck/Back	
6MWT	–	+ 75 / + 58	+ 60 / + 55	+ 83
SRT	–	NR (+ 25 to + 55) / +39	NR (+ 25 to + 50) / + 44	+61
1MSCT	–	+ 18 / + 16	+ 15 / + 18	+ 25
STS	–	NR (−2.0 to −3.3) / -5	NR (−4.0 to −6.0) / -7	−6
JAM	+ 5 / + 6	–	–	+ 9
PILE	NR (+ 2.5 to + 5.0) / + 4	NR (0 to + 2.5) / + 4	NR (+ 2.5 to + 5.0) / + 4	+ 7

*NR* Not Relevant, only a range of values is given because no values are associated with both sensitivity and specificity ≥60% (the range boundaries correspond to the thresholds with either 60% sensitivity or 60% specificity). 6-Minute Walk Test (6MWT) in meters, Steep Ramp Test (SRT) in Watts, 1-Minute Stair Climbing Test (1MSCT) in steps, Sit-To-Stand test (STS) in seconds, Jamar dynamometer test (JAM) in Newtons, Progressive Isoinertial Lifting Evaluation (PILE) in kg

**Table 4:** Correlation coefficients between the GRC (− 3 to + 3) and changes in scores (Δ = follow up score – baseline score)

Changes in Scores	Upper limb	Lower limb	Neck/Back
Δ 6MWT	–	0.351^a^	0.372^a^
Δ SRT	–	0.220^a^	0.184
Δ 1MSCT	–	0.424^a^	0.331^a^
Δ STS	–	0.067	−0.046
Δ JAM	0.338^a^	–	–
Δ PILE	0.312^a^	0.241^a^	0.171

6MWT: 6-Minute Walk Test, *SRT* Steep Ramp Test, *1MSCT* 1-Minute Stair Climbing Test, *STS* Sit-To-Stand test, *JAM* Jamar dynamometer test, *PILE* Progressive Isoinertial Lifting Evaluation. ^a^Statistically significant

### MCID - distribution-based and opinion-based methods

SEM values for each functional score according to lesion location are also reported in Table 3, as are the Round 3 Delphi results. For all tests except the SRT, the estimations made by physiotherapists and occupational therapists were 25–83% higher than those made by physicians (96 m/58w/27 steps/11 kg/8 kg, vs. 60 m/69w/21 steps/6 kg/6 kg), respectively.

## Discussion

Using the Anchor-based method, the MCIDs of the 6MWT and 1MSCT were estimated at + 75 m / + 18 steps (lower-limb lesions) and + 60 m / + 15 steps (neck-back lesions) and the MCID of the JAM was estimated at + 5 kg (upper-limb lesions). For the SRT, STS and PILE, only distribution-based and opinion-based estimations were relevant (sensitivity and specificity criterion not met for the anchor method): + 39w to + 61w, − 5 s to − 7 s and + 4 kg to + 7 kg, depending on the method and the lesion location. The above values may be used as a basis for interpreting clinical changes in a patient or in a group of patients participating in a therapeutic trial, and in determining the minimal sample size in a new trial.

Distribution-based and Anchor-based estimations were close to each other in many cases. This is consistent with previous studies, in which MCIDs based on patients’ global rating as the anchor were close to the value of one SEM [33, 34]. On the other hand, most MCIDs in the opinion-based method were higher than those in the other methods. This was mainly due to the more optimistic judgment of therapists as the functional improvement they expected was greater than that expected by the physicians and by the patients themselves. Another hypothesis is that our physicians had an overall view of the patients across the different therapies and over time, including before and after the rehabilitation program, whereas our therapists focused on their own domain and were less familiar with the long-term follow-up. Physicians may also have been more aware than our therapists about previously published values of MCID, and could have been influenced by the literature. For example, MCIDs for the 6MWT did not exceed 25 m in a previous study on cardiac rehabilitation, in which one of the authors of the present article took part [11]. We can speculate that the other physicians on the opinion team were not inclined to set an MCID as high as 96 m, the improvement set by the therapists.

Some authors have argued that the anchor-based approach is the best choice because it is the only method that addresses the patient’s perspective. In a recent review of the literature on MCID estimations for the 6MWT, Bohannon et al. [20] only took into consideration articles using this particular approach and excluded articles reporting procedures other than the ROC analysis, as recommended by Terwee et al. [22]. For Turner et al., distribution-based indicators should only be used “*as temporary substitutes pending availability of empirically established anchor-based indicators*” [21]. On the other hand, the MCID should be at least equal to the SEM, which measures the variation due to unreliability [23]. For all of the above reasons, the highest MCIDs for the Anchor-based and Distribution-based estimations should be considered acceptable: + 75 m and + 60 m for the 6MWT (lower-limb and neck/back lesions, respectively), + 18 steps and + 18 steps for the 1MSCT (lower-limb and neck/back lesions, respectively) and + 6 kg for the JAM.

Our results showed that the lesion location also affected MCIDs. This result was not surprising at all because walking performance is more severely impaired in patients with lower-limb lesions than in those with neck/back lesions, and the former obviously expect a greater improvement in function directly related to their lesion. As anticipated, MCID estimations differed from previously published values in different groups of patients. For example, MCIDs of the 6MWT have been estimated at 20 m for patients with Duchenne muscular dystrophy [13] and at 25 m in patients with coronary artery disease [11], which is far less than the present value in the lower-limb group. However, it is not surprising to note that the expected improvement in walking performance is lower in boys with a Duchenne muscular dystrophy than in chronic musculoskeletal pain patients. The low MCID value in coronary artery patients could be explained by pathological or psychological factors. The choice of a different anchor could also partly explain this difference (same limit at 2, but 9-level vs. 7-level GRC in the present study). Different MCID values for the 6MWT in different settings are available in Bohannon et al. [20]. The MCID of the PILE was estimated at + 2.5 kg in chronic back pain patients completing a functional restoration program [14], which is a bit less than our 4 kg. This difference could be explained by the great proportion of men in our cohort (88% vs. 59% in Gatchel [14]). The MCID of STS was previously estimated at 1.7 s in patients with chronic obstructive pulmonary disease and at 2.3 s in vestibular rehabilitation [35, 36], which is far less than in our patients. In the first case, patients’ performances at baseline were as good as our patients’ performances at discharge. Therefore, the potential for improvement was lower. In the second case, the presence of neurological balance disorders may explain a relatively small MCID value. It was estimated for the JAM at 5.0–6.2 kg in stroke and 6.5 kg in distal radius fracture, which is close to our estimations, and to 0.84 kg in a population of 70 to 90 year old females with thumb carpometacarpal osteoarthritis, which is much less than in our population of mostly male blue-collar workers [37–39].

Parameters other than the disease may influence MCID estimations. For instance, the MCID for the JAM in stroke patients was lower if the motor deficiency affected the dominant hand (5 kg) rather than the non-dominant hand (6.2 kg) [37], and the MCID for the 6MWT varies over the course of the disease, as is the case in subacute or chronic stroke (61 vs. 34.4 m) [40].

It is therefore crucial to estimate MCIDs in homogeneous groups of patients, and our very large population made it possible to produce separate MCID estimations for chronic musculoskeletal pain patients with lower-limb, upper-limb and neck/back lesions.

This is the first study to attempt to estimate the MCID of six functional tests simultaneously and separately in patients with upper-limb, lower-limb and neck/back lesions. We used three different approaches, including the anchor-based approach with ROC analysis, which is considered the reference method by some authors [19–22]. Although we believe that selecting anchors that are specific to each performance test could have led to slightly different results, our global anchor GRC was validated a posteriori for the 6MWT, 1MSCT and JAM, as it was found to correlate strongly enough with changes in these scores (*r* > 0.3). GRC scores also correlated strongly with changes in mood and pain scales, suggesting that pain and psychological improvements also contributed to patients’ satisfaction. Conversely, GRC scores correlated less strongly with scores improvement of SRT, STS and PILE, but we can assume that the MCIDs might have been estimated for these three scales with anchors specifically related to strength or endurance.

### Study limitations

The main limitation of this study was a potential selection bias because all of our patients were of working age (18–65), mostly men and presented with lesion in only one area (neck/back, upper limb or lower limb). It is more than likely that MCID estimations would have been slightly lower in an older population or might have differed in a predominantly female population. Another limitation is that we cannot guaranty that all patients specifically answered the GRC with respect to the lesion injury they were hospitalized for. For example, a patient presenting with a chronic low back pain and comes to rehabilitation for a leg injury could be influenced by the back pain when completing the GRC. If we had not included this kind of patients, we might have estimated more MCID values with the ROC technique, but we would have deviated from real-life conditions.

Finally, we are aware that our way of evaluating the SEM is not ideal, because it is based on repeated measures with an intervention in between, but no data were available with repeated evaluations within a short time period. Moreover, it was not possible to implement such repeated evaluations in the therapeutic program of the patients, which is already very dense. However, our estimation is not penalized by a systematic bias (which is here due to the effect of the intervention, and not to a lack of reliability) and is in line with Rousson’s recommendations [41].

Tangent to this debate, and importantly, one should remember that the MCID is of limited value in the management of individuals taking part in multidisciplinary rehabilitation programs. By way of illustration, let us say that + 5 kg is a good estimation of the MCID for the JAM in patients with painful upper limb lesions. This only means that most patients who are satisfied with their clinical outcome after a multidisciplinary rehabilitation program have improved their grip strength by at least + 5 kg in this test. Now let us imagine that the main focus of a new rehabilitation program is grip strength to the detriment of other dimensions, like hand dexterity, bimanual activities, pain management, psychological support, etc. A high percentage of patients who reached this grip-strength MCID could be “globally unsatisfied” as their objectives in the other aspects were not met. This leads us to the opinion that the present estimations of MCIDs are not tailored for “all chronic musculoskeletal pain patients”, but for “chronic musculoskeletal pain patients participating in a vocational oriented multidisciplinary rehabilitation program”, where grip strength, walking speed or climbing stairs are only three aspects of many. In other words, these values should not be used to set goals at an individual level, unless the patient explicitly expresses a wish to increase grip strength, or walk or climb stairs faster.

## Conclusion

MCIDs were estimated at + 75 m and + 60 m for the 6MWT (lower-limb and neck/back lesions, respectively), + 18 steps for the 1MSCT (lower-limb and neck/back lesions) and + 6 kg for the JAM (upper limb lesions). Distribution and opinion-based methods provided rough estimations of MCIDs for the SRT (+ 39w to + 61w), the STS (− 5 s to -7 s) and the PILE (+ 4 kg to + 7 kg). The use of specific anchors might eventually give better estimations for these three scales. Future research is needed to estimate MCIDs for functional scales in older chronic musculoskeletal pain patients.

## Abbreviations

1MSCT: 1-min stair climbing test
6MWT: 6-min walk test
GRC: Global Rating of Change
JAM: Jamar dynamometer test
MCID: Minimal Clinically Important Difference
PILE: Progressive Isoinertial Lifting Evaluation
SEM: Standard Error of Measurement
SRT: Steep Ramp Test
STS: Sit-to-stand test

## References

[1] Sugai K (2017). Chronic musculoskeletal pain in Japan (the final report of the 3-year longitudinal study): association with a future decline in activities of daily living. J Orthop Surg (Hong Kong).

[2] Vos, T., et al., Years lived with disability (YLDs) for 1160 sequelae of 289 diseases and injuries 1990-2010: a systematic analysis for the global burden of disease study 2010*.* Lancet, 2012. 380(9859): p. 2163–96. 10.1016/S0140-6736(12)61729-2 PMC6350784 23245607

[3] http://www.nfp53.ch/files/download/nfp53_synthesebericht_1004_f.pdf Programme national de recherche PNR 53. Santé musculo-squelettique – douleurs chroniques. Rapport de synthèse du Comité de direction. Octobre 2009.

[4] Butland RJ (1982). Two-, six-, and 12-minute walking tests in respiratory disease. Br Med J (Clin Res Ed).

[5] De Backer IC, et al. Exercise testing and training in a cancer rehabilitation program: the advantage of the steep ramp test. Arch Phys Med Rehabil. 2007;88(5):610–6. 10.1016/j.apmr.2007.02.013 17466730

[6] Macnicol MF, Uprichard H, Mitchell GP (1981). Exercise testing after the Chiari pelvic osteotomy. J Bone Joint Surg Br.

[7] Bohannon RW (1995). Sit-to-stand test for measuring performance of lower extremity muscles. Percept Mot Skills.

[8] Mathiowetz V (1984). Reliability and validity of grip and pinch strength evaluations. J Hand Surg Am.

[9] Mayer TG (1988). Progressive isoinertial lifting evaluation. I. A standardized protocol and normative database. Spine (Phila Pa 1976).

[10] Jaeschke R, Singer J, Guyatt GH (1989). Measurement of health status. Ascertaining the minimal clinically important difference. Control Clin Trials.

[11] Gremeaux V (2011). Determining the minimal clinically important difference for the six-minute walk test and the 200-meter fast-walk test during cardiac rehabilitation program in coronary artery disease patients after acute coronary syndrome. Arch Phys Med Rehabil.

[12] Crapo RO (2002). ATS statement: guidelines for the six-minute walk test. Am J Respir Crit Care Med.

[13] McDonald CM (2013). The 6-minute walk test and other clinical endpoints in duchenne muscular dystrophy: reliability, concurrent validity, and minimal clinically important differences from a multicenter study. Muscle Nerve.

[14] Gatchel RJ (2013). Validation of a consensus-based minimal clinically important difference (MCID) threshold using an objective functional external anchor. Spine J.

[15] Make B (2007). How can we assess outcomes of clinical trials: the MCID approach. COPD.

[16] Wang YC, et al. Global rating of change: perspectives of patients with lumbar impairments and of their physical therapists. Physiother Theory Pract. 2018:1–9. 10.1080/09593985.2018.1458930 29608121

[17] Katz NP, Paillard FC, Ekman E (2015). Determining the clinical importance of treatment benefits for interventions for painful orthopedic conditions. J Orthop Surg Res.

[18] Revicki D (2008). Recommended methods for determining responsiveness and minimally important differences for patient-reported outcomes. J Clin Epidemiol.

[19] Devji T (2017). Application of minimal important differences in degenerative knee disease outcomes: a systematic review and case study to inform BMJ Rapid Recommendations. BMJ Open.

[20] Bohannon RW, Crouch R (2017). Minimal clinically important difference for change in 6-minute walk test distance of adults with pathology: a systematic review. J Eval Clin Pract.

[21] Turner D (2010). The minimal detectable change cannot reliably replace the minimal important difference. J Clin Epidemiol.

[22] Terwee CB (2007). Quality criteria were proposed for measurement properties of health status questionnaires. J Clin Epidemiol.

[23] Copay AG (2007). Understanding the minimum clinically important difference: a review of concepts and methods. Spine J.

[24] Wells G (2007). Determining the minimal clinically important differences in activity, fatigue, and sleep quality in patients with rheumatoid arthritis. J Rheumatol.

[25] Palmer CS, Gabbe BJ, Cameron PA (2016). Defining major trauma using the 2008 Abbreviated Injury Scale. Injury.

[26] Cleeland CS, Ryan KM (1994). Pain assessment: global use of the Brief Pain Inventory. Ann Acad Med Singap.

[27] Zigmond AS, Snaith RP (1983). The hospital anxiety and depression scale. Acta Psychiatr Scand.

[28] Kamper SJ, Maher CG, Mackay G (2009). Global rating of change scales: a review of strengths and weaknesses and considerations for design. J Man Manip Ther.

[29] Andersson EI, Lin CC, Smeets RJ (2010). Performance tests in people with chronic low back pain: responsiveness and minimal clinically important change. Spine (Phila Pa 1976).

[30] Juniper EF (1994). Determining a minimal important change in a disease-specific Quality of Life Questionnaire. J Clin Epidemiol.

[31] Weir JP (2005). Quantifying test-retest reliability using the intraclass correlation coefficient and the SEM. J Strength Cond Res.

[32] Nu*mber Cruncher Statistical System (9).* 2013: Atlanta.

[33] Norquist JM, Fitzpatrick R, Jenkinson C (2004). Health-related quality of life in amyotrophic lateral sclerosis: determining a meaningful deterioration. Qual Life Res.

[34] Wyrwich KW (1999). Linking clinical relevance and statistical significance in evaluating intra-individual changes in health-related quality of life. Med Care.

[35] Jones SE (2013). The five-repetition sit-to-stand test as a functional outcome measure in COPD. Thorax.

[36] Meretta BM (2006). The five times sit to stand test: responsiveness to change and concurrent validity in adults undergoing vestibular rehabilitation. J Vestib Res.

[37] Lang CE (2008). Es*timating minimal clinically important differences of upper-extremity measures early after stroke*. Arch Phys Med Rehabil.

[38] Kim JK, Park MG, Shin SJ (2013). What is the Minimum Clinically Important Difference in Grip Strentgh. Clin Orthop Relat Res.

[39] Villafane JH, et al. Minimal Clinically Important Difference of Grip and Pinch Strength in Women With Thumb Carpometacarpal Osteoarthritis When Compared to Healthy Subjects. Rehabil Nurs. 2014. 10.1002/rnj.196 25557054

[40] Louie DR, Eng JJ (2016). Powered robotic exoskeletons in post-stroke rehabilitation of gait: a scoping review. J Neuroeng Rehabil.

[41] Rousson V, Gasser T, Seifert B (2002). Assessing intrarater, interrater and test-retest reliability of continuous measurements. Stat Med.

